# Preparation and Characterization of Films Based on a Natural P(3HB)/mcl-PHA Blend Obtained through the Co-culture of *Cupriavidus Necator* and *Pseudomonas Citronellolis* in Apple Pulp Waste

**DOI:** 10.3390/bioengineering7020034

**Published:** 2020-04-05

**Authors:** Ana Teresa Rebocho, João R. Pereira, Luísa A. Neves, Vítor D. Alves, Chantal Sevrin, Christian Grandfils, Filomena Freitas, Maria A. M. Reis

**Affiliations:** 1 UCIBIO-REQUIMTE-Applied Molecular Biosciences Unit Chemistry Department, New University of Lisbon, 1099-085 Lisboa, Portugal; at.rebocho@campus.fct.unl.pt (A.T.R.); jra.pereira@campus.fct.unl.pt (J.R.P.); amr@fct.unl.pt (M.A.M.R.); 2 LAQV-REQUIMTE–Laboratório Associado para a Quimica Verde Chemistry Department, New University of Lisbon, 1099-085 Lisboa, Portugal; lan11892@fct.unl.pt; 3 LEAF, Linking Landscape, Environment, Agriculture and Food, Instituto Superior de Agronomia, University of Lisbon, 1649-004 Lisboa, Portugal; vitoralves@isa.ulisboa.pt; 4 CEIB-Interfaculty Research Centre of Biomaterials, University of Liège, 4000 Liège, Belgium; csevrin@uliege.be (C.S.); c.grandfils@uliege.be (C.G.)

**Keywords:** apple pulp waste, polyhydroxyalkanoates (PHA), co-culture, polymer blend, films

## Abstract

The co-culture of *Cupriavidus necator* DSM 428 and *Pseudomonas citronellolis* NRRL B-2504 was performed using apple pulp waste from the fruit processing industry as the sole carbon source to produce poly(3-hydroxybutyrate), P(3HB) and medium-chain length PHA, mcl-PHA, respectively. The polymers accumulated by both strains were extracted from the co-culture’s biomass, resulting in a natural blend that was composed of around 48 wt% P(3HB) and 52 wt% mcl-PHA, with an average molecular weight of 4.3 × 10^5^ Da and a polydispersity index of 2.2. Two melting temperatures (T_m_) were observed for the blend, 52 and 174 °C, which correspond to the T_m_ of the mcl-PHA and P(3HB), respectively. P(3HB)/mcl-PHA blend films prepared by the solvent evaporation method had permeabilities to oxygen and carbon dioxide of 2.6 and 32 Barrer, respectively. The films were flexible and easily deformed, as demonstrated by their tensile strength at break of 1.47 ± 0.07 MPa, with a deformation of 338 ± 19% until breaking, associated with a Young modulus of 5.42 ± 1.02 MPa. This study demonstrates for the first time the feasibility of using the co-culture of *C. necator* and *P. citronellolis* strains to obtain a natural blend of P(3HB)/mcl-PHA that can be processed into films suitable for applications ranging from commodity packaging products to high-value biomaterials.

## 1. Introduction

Polyhydroxyalkanoates (PHAs) are natural polyesters accumulated intracellularly by a variety of bacteria and Archaea, as carbon and energy storage compounds. These biopolymers have attracted great attention because they are obtained from renewable resources, are biodegradable and biocompatible, and present excellent material properties that range from rigid thermoplastics to flexible elastomers [1]. Short-chain-length PHAs (scl-PHAs), which are composed of monomers with 3 to 5 carbon units, are mostly rigid and brittle thermoplastics, characterized by high crystallinity degrees and high melting temperatures, while medium-chain-length PHAs (mcl-PHAs) that contain units with 5 to 14 carbons are more elastic and/or viscous materials, characterized by low crystallinity degrees, glass transition temperatures, and melting temperatures [2]. Moreover, the material properties of PHAs can be tailored by choosing the appropriate producing bacteria and substrates [2,3]. Given this set of characteristics, PHAs hold huge potential for use in a wide range of applications, including not only packaging but also medical applications (such as in drug delivery or tissue engineering) [3,4]. The use of PHAs in the medical field is supported by the fact that in vivo, their degradation products, monomeric and oligomeric molecules, exert no toxic nor negative effects to human or animal cells or tissue [3,5].

Many of PHAs’ applications rely on their ability to form stand-alone films. Such films display mechanical properties and gas barrier properties that render them suitable for use in food packaging, hygiene, and medical film products [4,5,6,7]. The PHAs films’ properties depend on the polymer composition and molecular mass distribution, as well as on the processing conditions [7]. Films made of poly(3-hydroxybutyrate), P(3HB), the most widely known PHA, are brittle and rigid, while those of mcl-PHA are more ductile and flexible [3,8]. 

The properties of PHAs films can further be tailored via blending with other PHAs or with different materials, namely other natural biodegradable polymers (e.g., polycaprolactone, polylactic acid or polysaccharides) or non-degradable synthetic polymers (e.g., polyvinyl alcohol, low- and high-density polyethylene) [7,9,10,11]. In the last years, the blending of PHAs with such polymers became an approach to improve the physical properties of P(3HB) [12], specifically its inherent brittleness by blending with other polymers, as well as to reduce the production costs of PHA [13]. The elongation at break of poly(hydroxybutyrate-co-hydroxyvalerate), P(HB-co-HV), was improved, adopting a mcl-PHA blend made in solution and composed of 41.6% mol/mol of 3-hydroxyoctanoate, 35.9% mol/mol of 3-hydroxydecanoate, and 22.5% mol/mol of 3-hydroxydodecanoate [13]. The thermal stability and mechanical properties of P(3HB) were improved by mixing under melt compounding an mcl-PHA composed of 8 mol% of 3-hydroxyoctanoate and 2 mol% of 3-hydroxyhexanoate [14].

An alternative strategy to obtain PHAs blends is the co-culturing of two or more bacterial strains able to produce different types of PHAs from the same substrate. This has been attempted by Ashby et al. [15] using *Pseudomonas oleovorans* NRRL B-14682 and *Pseudomonas corrugata* 388 to convert glycerol into P(3HB)/mcl-PHA natural blends with adjustable blend ratios. This approach has the clear advantage of saving costs related to polymer extraction and purification, as the PHAs are extracted together from the same biomass in a single procedure, instead of dealing with their individual extractions.

Despite their proven valuable properties and the demonstrated suitability of different PHAs for a wide range of applications, the industrial development of these biopolymers is still hindered by the high production costs of most proposed bioprocesses. Feedstock price is one of the major issues, accounting for up to 30% of the overall production costs of PHAs [16]. The use of low-cost feedstocks, such as, for example, food wastes, agriculture and industrial wastes/by-products, and fruit and vegetable processing wastes, among others, have been proposed as a strategy to overcome this problem [17,18]. In this context, fruit processing wastes arise as valuable feedstocks due to their high sugar contents that can be utilized by many bacteria for cell growth and/or PHA synthesis [19,20].

In this study, the co-culture of *Cupriavidus necator* DSM 428 and *Pseudomonas citronellolis* NRRL B-2504 was performed using apple pulp waste from the fruit processing industry as the sole carbon source to produce P(3HB) and mcl-PHA, respectively. The polymers accumulated intracellularly by both strains were extracted from the co-culture biomass, resulting in a natural blend that was characterized in terms of its composition, molecular mass distribution, and thermal properties. P(3HB)/mcl-PHA blend films were prepared and their permeability to oxygen and carbon dioxide, water contact angle, swelling behavior, and mechanical properties were determined.

## 2. Materials and Methods 

### 2.1. Microorganisms and Media

*Pseudomonas citronellolis* NRRL B-2504 was kindly offered by the Agricultural Research Service of the Northern Regional Research Laboratory (ARS-NRRL), USA. *Cupriavidus necator* DSM 428 was purchased from the German Collection of Microorganisms and Cell cultures (DSMZ), Germany. Both microorganisms were preserved in glycerol (20%, *v/v*) (99%, Sigma-Aldrich, St. Louis, MO, USA), as a cryoprotectant agent, at −80 °C. 

Luria-Bertani (LB) broth (bacto tryptone, 10 g/L; yeast extract, 5 g/L; NaCl, 10 g/L; pH7) (99%, PancReac) was used for culture reactivation from the cryopreserved stocks and for inocula preparation. For the bioreactor experiments, the apple pulp waste was treated as described by Rebocho et al. [21]. Briefly, the apple pulp was mixed with deionized water (1:3, *v/v*), for viscosity reduction, and centrifuged (7012 g, 30 min). The insoluble solids were discarded, and the sugar-rich supernatant was sterilized by autoclaving at 121 °C and 1 bar, for 30 min. After cooling, the apple pulp extract was supplemented with 1 L of a mineral salts solution composed of (NH_4_)_2_HPO_4_, 2.2 g/L; K_2_HPO_4_, 11.6 g/L; KH_2_PO_4_, 7.4 g/L, 100 mL of 100 mM of MgSO_4_ solution, and 10 mL of micronutrient solution. The micronutrients solution contained the following (per L of 1 N HCl): FeSO_4_·7H_2_O, 2.78 g; MnCl_2_·4H_2_O, 1.98 g; CoSO_4_·7H_2_O, 2.81 g; CaCl_2_·2H_2_O, 1.67 g; CuCl_2_·2H_2_O, 0.17 g; ZnSO_4_·7H_2_O, 0.29 g. All reagents were acquired with 99% purity from Sigma-Aldrich (USA).

### 2.2. Bioreactor Cultivation

*P. citronellolis* NRRL B-2504 and *C. necator* DSM 428 were co-cultured in a bioreactor with a working volume of 10 L (BioStat B-Plus, Sartorius, Germany), with a starting volume of 9.5 L. The cultivation runs were initiated by inoculating 400 mL of each culture grown in LB medium for 24 h, at 30 °C and 200 rpm, in an orbital shaker.

The temperature and the pH were controlled at 30.0 ± 0.1 °C and 7.00 ± 0.02, respectively. The pH was controlled by the automatic addition of 5 M NaOH (98%, Sigma-Aldrich, USA) or 2 M HCl (37%, Sigma-Aldrich, USA). A constant air flow rate (4 SLPM, standard liters per minute) was kept during the cultivation runs and the dissolved oxygen concentration (DO) was controlled at 30% of the air saturation by the automatic adjustment of the stirring speed (300–800 rpm). Foam formation was suppressed by the automatic addition of Antifoam A (Sigma-Aldrich, USA). The bioreactor was operated under a batch mode during 48 h. Samples (24 mL) were collected from the bioreactor for biomass, PHA, and nutrient quantification. 

A bioreactor cultivation was also performed, under similar conditions, with a monoculture of *C. necator* for comparison.

### 2.3. Analytical Techniques

For determination of the cell dry mass (CDM), the cell pellets, obtained by centrifugation of the culture broth (10,956g, 15 min, 4 °C), were washed with deionized water and lyophilized. The CDM was determined by weighing the dried cell pellets.

PHA content and composition were determined by gas chromatography (GC) following the methanolysis of the lyophilized biomass samples, using the method described by Rebocho et al. [21]. The obtained methyl esters were analyzed in a Restek column (Crossbond, Stabilwax) at a constant pressure (96 kPa) using helium as the carrier gas. Splitless mode injection was used. The oven temperature program was the following: 20°C min^−1^ until 100 °C; 3 °C min^−1^ until 155 °C; and, finally, 20 °C min^−1^ until 220 °C.

A calibration curve for 3-hydroxyhexanoate (3HHx), 3-hydroxyoctanoate (3HO), 3-hydroxydecanoate (3HD), 3-hydroxydodecanoate (3HDd), and 3-hydroxytetradecanoate (3HTd) was made using home-made mcl-PHA with the following composition, validated by GC-MS (GC-Agilent 6890N (California, USA); MS-Thermo DSQ (Florida, USA): 3 mol% 3HHx, 17 mol% 3HO, 57 mol% 3HD, 11 mol% 3HDd, and 12 mol% 3HTd, at concentrations ranging from 0.1 to 2.0 g/L. For 3-hydroxybutyrate (3HB) and 3-hydroxyvalerate (3HV) monomers, a calibration curve was made using a copolymer of P(3HB-co-3HV) (Sigma-Aldrich, 88 mol% 3HB, 12 mol% 3HV), at concentrations ranging from 0.3 to 3.0 g/L. The obtained methyl esters were analyzed in a Restek column (Crossbond, Stabilwax) coupled with a flame ionization detector (FID), as described by Rebocho et al. [21].

The polymer content in the biomass (%PHA, wt%) was calculated using the following equation:

(1) $$\%PHA=\frac{\left[PHA\right]}{CDM}\times 100$$

where [*PHA*] is the polymer concentration (g/L), determined by GC.

The sugars’ (glucose, fructose, sucrose) quantification in the cell-free supernatant samples was performed by high performance liquid chromatography (HPLC) using a VARIAN Metacarb 87H column, coupled to a refractive index (RI) detector, as described by Rebocho et al. [21]. Glucose (Fluka, 99%), fructose (Scharlau, 99%), and sucrose (Fluka, 99%) were used as standards, at concentrations ranging from 0.0625 to 1.0 g/L. The ammonium concentration was determined by colorimetry using a flow segmented analyzer (Skalar 5100, Skalar Analytical, Breda, The Netherlands). All measurements were done in replicate analyses.

For determination of the total nitrogen, a kit (LCK 388, LATON^®^, Manchester, UK) with a detection range of 20–100 mg/L was used, as described by Rebocho et al. [21].

### 2.4. Biopolymer Extraction and Purification

At the end of the assays, the cultivation broth was recovered from the bioreactor and centrifuged (13,131 g, for 20 min, 4 °C). The cell pellets thus obtained were resuspended in deionized water and centrifuged again under the same conditions. The washed biomass was then lyophilized for 48 h. The PHA was extracted from the dried biomass by Soxhlet extraction with chloroform (Sigma-Aldrich, USA, 99.5%), at 80 °C for 48 h, and purified by precipitation in ice-cold ethanol (1:10, *v/v*) under vigorous stirring, as described by Pereira et al. (2019) [22].

### 2.5. Blend Characterization 

#### 2.5.1. P(3HB) and mcl-PHA Contents

To evaluate the content of each type of polymer, P(3HB) and mcl-PHA, in the blend obtained from the co-culture bioreactor assays, the method described by Ashby et al. [15] was used, with some modifications. In brief, 1.0 g of the polymer blend was mixed with 30 mL of acetone in a flask that was kept at 30 °C for 24 h, under constant agitation (150 rpm) in an orbital shaker. The acetone-soluble polymer fraction, containing mcl-PHA, was then separated from the acetone-insoluble polymer fraction, containing P(3HB), by centrifugation (11,180× *g*, 10 min, 10 °C). Each fraction was placed into dry pre-weighed vials, and left in a fume hood at room temperature for complete solvent evaporation.

#### 2.5.2. PHA Composition

The PHA monomer composition of the blend and each polymer fraction recovered from the acetone fractionation was determined by GC, as described above, but using a lower amount of sample (~3 mg).

#### 2.5.3. Fourier Transform Infrared Spectroscopy

Fourier transform infrared spectroscopy (FTIR) analysis was conducted with a Perkin-Elmer Spectrum two spectrometer. The polymer was directly analyzed on the FTIR cells. The spectra were recorded between 400 and 4000 cm^−1^ resolution with 10 scans, at room temperature.

#### 2.5.4. Molecular Mass Distribution

The number average molecular weight (Mn), weight average molecular weight (Mw), and polydispersity index (Mw/Mn) of polyesters were measured by size exclusion chromatography (SEC) as described by Rebocho et al. [21]. In brief, SEC was performed in chloroform at 30 °C adopting a Waters Millenium equipment. A 15-mg sample was dissolved in 3 mL of chloroform. Chromatographic separation was realized at a flowrate of 1 mL/min on Polymer Laboratories analytical columns (300 × 7.5 mm PLgel 5 µm 10^4^ Å; PLgel 5 µm 500 Å protected by a guard column). A refractive index detector was used (Waters, model 2410). Relative molecular weights of the polymers were determined according to the universal calibration method adopting standard polystyrene with molecular weight between 800 and 504,500 and using Water Millenium SEC software.

#### 2.5.5. Thermal Properties

The thermal properties of the biopolymer samples were determined by differential scanning calorimetry (DSC) and thermogravimetric analysis (TGA). TGA was performed using a thermogravimetric equipment Labsys EVO (Setaram, France). The sample were placed in an aluminum pan and analyzed at a heating rate of 10 °C/min in a temperature ranging between 25 and 500 °C. DSC was performed using a differential scanning calorimeter Discovery Series DSC25 (TA Instruments, New Castle, DE, USA) coupled to a cooling system 90 (TA Instruments Refrigerated Cooling System 90, USA). The samples were placed in an aluminum pan and analyzed in the temperature range between −90 and 100 °C for mcl-PHA polymer and a range between −90 and 200 °C for PHB, with heating and cooling steps of 10 °C min^−1^ under a nitrogen atmosphere. Three heating cycles were performed.

The glass transition temperature (T_g_, °C) was taken as the midpoint of the heat flux step, and the melting temperature (T_m_, °C) was determined at the minimum of the endothermic peak. The thermal degradation temperature (T_deg_, °C) corresponds to the temperature value obtained for the maximum decreasing peak of the sample mass. 

#### 2.5.6. X-Ray Diffraction

The structural analysis of the biopolymer samples was performed by X-ray diffraction (XRD), as described by Rebocho et al. [21], using a X’Pert Pro X-ray diffractometer from PANalytical (Almelo, Netherlands), equipped with an X’Celerator detector, in a Bragg–Brentano geometry with Cu Kα line radiation (λ = 1.5406° A). The 2θ scans were performed from 10° to 90°, with a step size of 0.03°.

### 2.6. Preparation and Characterization of Films

#### 2.6.1. Films Preparation

PHA solutions were prepared by dissolving 1.0 g of mcl-PHA or P(3HB) in 20 mL of chloroform (HPLC grade, Sigma-Aldrich, USA), under constant stirring, at room temperature, until complete dissolution, as described by Rebocho et al. [21]. The blend films were prepared as described by Azari et al. [23], by dissolving the polymer (1.0 g) in 20 mL of a 1:9 (*v/v*) dimethylformamide and chloroform solution (HPLC grade, Sigma-Aldrich, USA), under constant stirring, at 60 °C during 24 h. The solutions were transferred into glass petri dishes (diameter of 10 cm), which were placed in a desiccator and kept at room temperature until complete solvent evaporation. Slow solvent evaporation was performed in a saturated chloroform atmosphere to avoid the formation of cracks and non-selective voids in the films and to guaranty their homogeneity.

#### 2.6.2. Morphological Characterization

The morphology of the obtained films was assessed by scanning electron microscopy using an energy dispersive spectroscope (SEM-EDS), as described by Rebocho et al. [21]. PHA films were placed in a desiccator until completely dry, frozen in liquid nitrogen, and fractured in small pieces, followed by coating with a thin layer of Au/Pd. The films were analyzed using an analytical JEOL 7001F scanning electron microscope (FEG-SEM, JEOL, USA Inc., Peabody, MA, USA) equipped with a field emission gun operated with an acceleration voltage of 15 kV. All samples were visualized on their surface and cross-section, using different amplifications.

#### 2.6.3. Water Contact Angles 

The contact angle of the films was measured by the sessile drop method, where a drop of distilled water was manually deposited on the films’ surface with a small syringe. The software CAM2008 (KSV Instruments Ltd, Helsinki, Finland) acquired 10 images per sample and the tangent was determined by fitting the drop shape to a known mathematical function. Multiple replicates were performed, and the mean angle was determined.

#### 2.6.4. Swelling in Water

Films samples with a size of 1.0 × 1.0 cm^2^ were weighed (Kern & Sohn GmbH, Balingen, Germany) and their thickness was measured with a micrometer (Elcometer, Manchester, UK). The samples were immersed in 15 mL of deionized water, in a closed vial, and kept at 30 °C during 24 h. The swelling degree in terms of the mass of the samples was calculated with the following equation:

(2) $$Swelling\;Degree=\frac{X2-X1}{X1}\times 100\%$$

where *X*1 and *X*2 are the initial and final mass (g) of the samples, respectively. After the immersion period (24 h), the films were cleaned with paper tissue and their thickness was measured with a micrometer (Elcometer, UK).

#### 2.6.5. Gas Permeation

Gas permeation tests for CO_2_ and O_2_ were conducted as described by Neves et al. [24]. The films’ permeability (*P*, Barrer) for pure CO_2_ and O_2_ gases was calculated according the following equation:

(3) $$\frac{1}{\beta }\times \ln\frac{\Delta_{p0}}{\Delta_{p}}=P\times \frac{t}{l}$$

where Δ*p* (bar) corresponds to the difference of the pressures in the feed and permeate compartments, *t* (s) is the time, and *l* (m) is the film’s thickness. *β* is a geometric parameter characteristic of the cell (m^−1^), and was obtained using Equation (4):

(4) $$\beta =A\left(\frac{1}{V_{feed}}+\frac{1}{V_{perm}}\right)$$

where *A* is the film’s area (cm^2^) and *V_feed_* and *V_perm_* are the volumes (bar) of the feed and permeate compartments, respectively. The gas permeability (*P*) was obtained from the slope when representing $\frac{1}{\beta }\ln\frac{\Delta_{p0}}{\Delta_{p}}$ as a function of $\frac{t}{l}$. In order to compare the results with those available in the literature, a conversion was made (1 Barrer = 1 × 10^−10^ cm^3^ (STP)·cm·cm^−2^·cmHg^−1^·s^−1^ = 8.3 × 10^−13^ m^2^·s^−1^) [25].

#### 2.6.6. Mechanical Properties

The films were cut into 2.5 × 1.5 cm rectangular-shaped strips, which had an average thickness of 100 µm, measured using a digital micrometer (Mitutoyo, Japan). Tensile tests were performed at room temperature (22 °C) at a deformation rate of 0.5 mm·s^−1^ using a TA-XT plus texture analyzer (Stable Micro Systems, Surrey, England) with a 5-kg load cell, as described by Rebocho et al. [21]. The samples’ Young modulus (MPa) was determined as the slope of the linear initial section of the stress–strain curve. The tensile stress at break (MPa) was calculated as the ratio of the maximum force to the films’ initial cross-sectional area. The deformation (strain) at break (-) was determined as the ratio of the extension of the sample upon rupture by the initial gage length. Three film replicas were analyzed.

## 3. Results and Discussion

### 3.1. Bioreactor Cultivation Experiments 

#### 3.1.1. *P. citronellolis* and *C. necator* Monocultures Experiments

**Figure 1:**
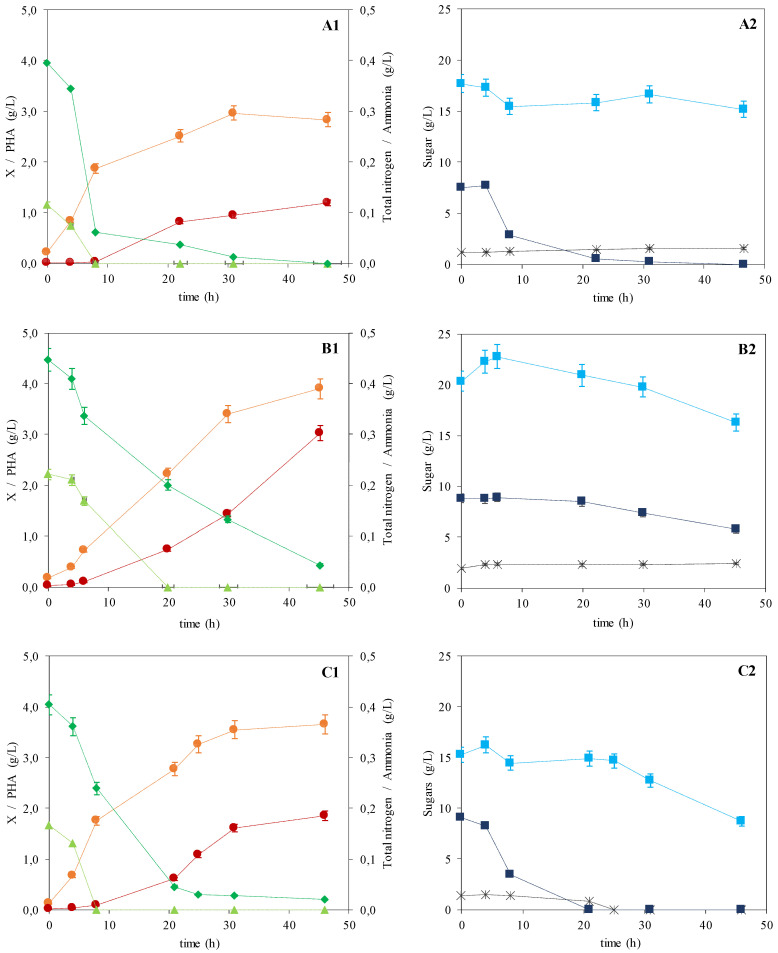
Cultivation profile for the monocultures of *P. citronellolis* (adapted from Rebocho et al. [21]) and *C. necator* (**A** and **B**, respectively), and the co-culture of *C. necator* and *P. citronellolis* (**C**), using apple pulp extract as the sole carbon source ((**1**): rest biomass, 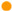; PHA, 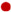; total nitrogen, 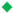; ammonia, 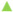; (**2**): glucose, 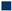; fructose, 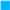; sucrose, 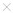; error bars correspond to duplicate analyses).

The apple pulp extract had a total sugars content of 31.09 ± 0.55 g/L, comprising 17.76 ± 2.53 g/L fructose, 8.48 ± 0.83 g/L glucose, 1.50 ± 0.40 g/L sucrose, and traces of arabinose (<0.5 g/L). This high content in simple sugars makes apple pulp an interesting feedstock for microbial cultivation. As reported by Rebocho et al. [21], *P. citronellolis* NRRL B-2504 was able to grow and accumulate PHA using apple pulp extract as the sole substrate (Figure 1A), reaching a CDM of 4.00 ± 0.08 g/L with a polymer content of 30.0 ± 1.7 wt% (Table 1), within 48 h of cultivation. However, while glucose was completely consumed, only 2.5 g/L of the initial fructose available were metabolized during the run (Figure 1(A2)). In contrast, the sucrose concentration did not change. These bioprocess conditions were therefore not optimal at all, leading to a large waste of the carbon source.

In order to improve the yield of this fermentation process, *C. necator* DSM 428 was selected for cultivation in apple pulp extract due to its known ability to utilize fructose as a carbon source while being unable to grow on glucose [26,27].

Upon cultivation on the apple pulp extract medium, *C. necator* was able to grow and synthesize PHA (Figure 1B). The results show that under such cultivation conditions, a final CDM of 6.93 ± 0.09 g/L was reached with a polymer content of 43.7 ± 2.5 wt% (Table 1). Polymer accumulation started at around 6 h of cultivation (Figure 1(B1)), attaining a final concentration of 3.03 ± 0.04 g/L, corresponding to an overall volumetric productivity of 0.066 ± 0.001 g/(L·h) (Table 1).

An overall sugar consumption of approximately 6.67 ± 0.25 g/L was noticed, corresponding to 4.07 ± 0.17 g/L fructose and 3.06 ± 0.08 g/L glucose (Figure 1(B2)). No sucrose consumption was detected, which is in accordance with literature reports [28]. The sugar concentration profiles show that fructose and glucose consumption was only initiated after 20 h of cultivation (Figure 1(B2)), despite the observed cell growth and polymer accumulation (Figure 1(B1)). Other nutrients (e.g., amino acids, vitamins) present in the apple pulp extract that may have served as substrate [29]. This assumption is supported by the fact that the total nitrogen content in the culture broth decreased concomitantly with cell growth during the initial 24 h of the run (Figure 1(B1)). From the 0.45 g/L total nitrogen available, 0.37 g/L corresponded to organic nitrogenous compounds (amino acids, peptides, vitamins) since an ammonia content of 0.22 g/L was detected in the extract. Such organic nitrogen sources have probably been used by the culture for cell growth and, also, polymer accumulation. The ability of *C. necator* strains to use organic nitrogen substrates (e.g., urea, corn steep liquor) for PHA production has been reported in studies reported earlier [30,31].

These results show that, although *C. necator* can produce PHA from apple pulp extract as the sole substrate, the sugar consumption is incomplete and, therefore, has poor efficiency in terms of bioprocessing.

#### 3.1.2. Co-Culture of *C. necator* and *P. citronellolis*

Following these results, a novel strategy was designed based on the co-culture of both strains given their differential preference over fructose and glucose as carbon sources. Hence, *P. citronellolis* NRRL B-2504 and *C. necator* DSM 428 were co-cultured, under similar cultivation conditions as the monocultures’ bioreactor runs (Figure 1C). As shown in Figure 1(C1), a CDM of 5.51 ± 0.09 g/L with an overall polymers’ content in the biomass of 33.6 ± 1.9 wt% was achieved (Table 1). From this data, an overall volumetric productivity of 0.040 ± 0.002 g/(L·h) was derived (Table 1). Compared to the monocultures (Figure 1A,B), the co-culture reached an active biomass concentration (3.66 ± 0.06 g/L) close to the value obtained for *C. necator* (3.90 ± 0.05 g/L) and higher than that of *P. citronellolis* (2.80 ± 0.06 g/L) (Table 1). The slightly lower value attained by the co-culture compared to the *C. necator* monoculture may have been due to the competition for nutrients (e.g., nitrogen, micronutrients) by both strains that might have induced some growth limitation. Despite ammonia’s depletion at around 8 h of cultivation, cell growth was supported until 30 h, probably due to the organic nitrogen resources (as shown by the total nitrogen content profile) (Figure 1(C1)). PHA production by the co-culture (1.85 ± 0.03 g/L) was within the values obtained for *P. citronellolis* and *C. necator* monocultures (1.20 ± 0.05 and 3.03 ± 0.04 g/L, respectively).

Glucose was exhausted within less than 20 h of cultivation and a fructose uptake of 6.59 ± 0.76 g/L was observed (Figure 1(C2)). In contrast with the monocultures’ assays, sucrose (1.34 ± 0.32 g/L) was also completely consumed. However, sucrose consumption was noticed only after glucose exhaustion. This ability to assimilate sucrose might have been triggered by glucose depletion, which did not occur during *C. necator* monoculture, in which glucose was available throughout the run (Figure 1(B2)). These figures corresponded to an overall sugars’ consumption of 17.02 ± 0.76 g/L, which is higher than the values obtained for either *P. citronellolis* or *C. necator* monocultures (10.03 ± 1.43 and 6.67 ± 0.25 g/L, respectively) (Table 1).

These results therefore support the co-culture strategy, being effective both in terms of sugar consumption from the apple pulp extract, and its conversion for polymer production.

### 3.2. Natural Blend Characterization

#### 3.2.1. P(3HB) and mcl-PHA Contents

The polymer samples extracted from the co-culture’s biomass comprised 48 wt% 3HB, 36 wt% 3HD, 10 wt% 3HO, 3 wt% 3HDd, 3 wt% 3HTd, and traces of 3HHx (0.5 wt%) (Table 2). The identified monomers encompass those of the individual polymers synthesized by each monoculture, namely *P. citronellolis* mcl-PHA and *C. necator* P(3HB). As reported by Rebocho et al. [21], under similar cultivation conditions, *P. citronellolis* produced a polymer composed of 68 wt% 3HD, 22 wt% 3HO, 5 wt% 3HDd, 4 wt% 3HTd, and 1 wt% 3HHx. On the other hand, *C. necator* synthesized a 3HB homopolymer (Table 2). The acetone fractionation procedure confirmed that the biopolymer blend was made of 52 and 48 wt% in mcl-PHA and P(3HB), respectively.

#### 3.2.2. Fourier Transform Infrared Spectroscopy

**Figure 2:**
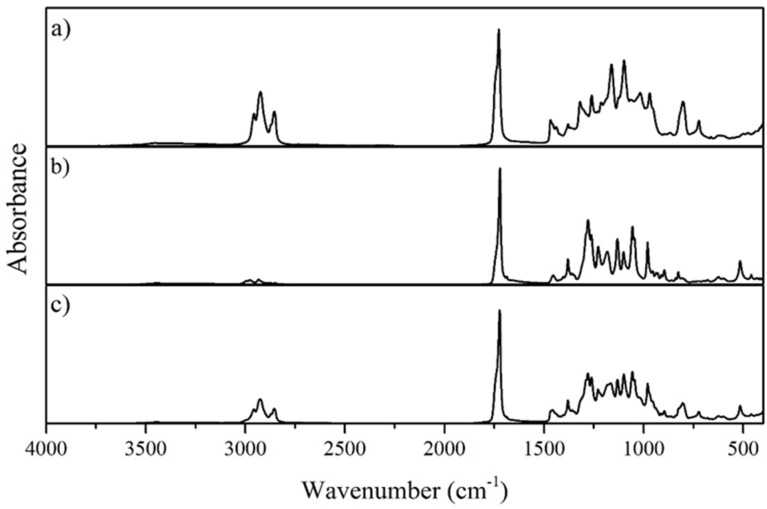
FTIR-ATR spectra of the mcl-PHA produced by *P. citronellolis* (**a**), the P(3HB) produced by *C. necator* (**b**), and the P(3HB)/mcl-PHA produced by the co-culture (**c**) from apple pulp waste.

The FTIR spectra (Figure 2) for both the mcl-PHA produced by *P. citronellolis* and the P(3HB) produced by *C. necator* show an intense absorption peak at 1727 cm^−1^ corresponding to the stretching band of the ester carbonyl group (C=O), which is also a characteristic of the crystalline phase [32]. This band is the strongest peak in the spectra, corresponding to a characteristic band of PHA.

Regarding the mcl-PHA FTIR spectrum (Figure 2a), near 2961–2854 cm^−1^, peaks can be identified that can be assigned to the stretching vibration due to asymmetric CH_2_ of the lateral monomeric chains and to the symmetrical methyl group [33]. Concerning the P(3HB) FTIR spectrum (Figure 2b), the weakest band in the spectra corresponds to the methylene C–H elongation vibration near 2900 cm^−1^, reported in the literature to be a vibration stronger for mcl-PHA and weaker regarding P(3HB). The bands between 1057 and 1278 cm^−1^ are related to the degree of crystallinity, with the 1057 cm^−1^ peak attributed to C–O bonds. The peaks obtained in the FTIR spectra for the P(3HB) produced by *C. necator* are comparable to those reported in the literature [34,35,36].

The FTIR analysis for the blend displays a group of similar peaks to those identified for mcl-PHA and P(3HB) separately (Figure 2c). The most intense absorption peak is located near 1723 cm^−1^ corresponding to the characteristic C=O peak in all the PHA FTIR spectrum [32]. Moreover, two peaks can be identified and assigned to the asymmetric CH_2_ of the lateral monomeric chains (the peak at 2924 cm^−1^) and the symmetrical methyl group (the peak at 2847 cm^−1^) [33]. Between 1000 and 1500 cm^−1^, the same group of peaks detected in the mcl-PHA and PHB FTIR spectrum is identified, indicating the presence of numerous characteristic structures within both polymers, namely the C–O bonds [34,35]. As it can been seen, the FTIR spectrum for the blend produced combine the peaks characteristic for mcl-PHA and P(3HB).

#### 3.2.3. Molecular Mass Distribution

The SEC analysis of the P(3HB)/mcl-PHA blend highlighted one peak, indicating that the P(3HB) and the mcl-PHA had identical M_w_ values. In fact, in the monocultures’ assays, *P. citronellolis* mcl-PHA had an M_w_ of 3.7 × 10^5^ Da, and that of *C. necator* P(3HB) was 5.0 × 10^5^ Da, while the blend had an M_w_ of 4.3 × 10^5^ Da (Table 2). Additionally, the PDI values were similar for all three samples within the range 2.0–2.2.

#### 3.2.4. Thermal Properties

**Figure 3:**
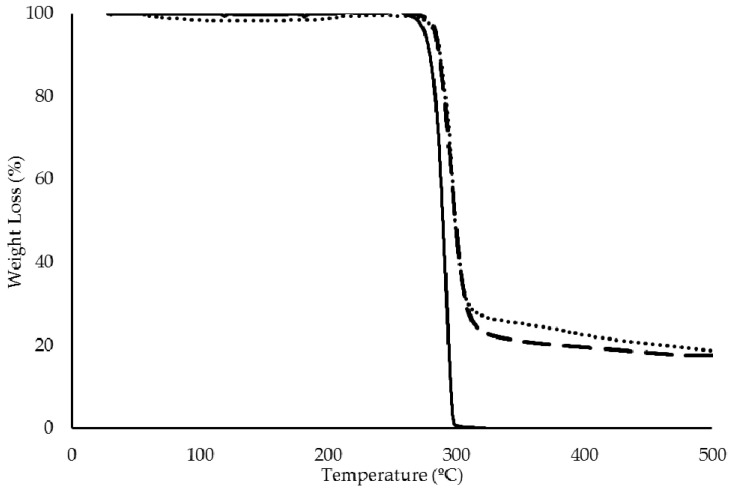
Thermogravimetric analysis (TGA) curves of pure P(3HB) (straight line **-**) and mcl-PHA (dotted line **∙∙∙**) produced by *C. necator* DSM 428 and *P. citronellolis* NRRL B-2504, respectively, and of their blend (dashed line **---**) isolated from the cultivation in apple pulp waste.

There was no detectable weight loss for the P(3HB)/mcl-PHA blend up to a temperature of 250 °C (Figure 3). The blend’s decomposition showed a single weight loss of approximately 98%, with a maximum degradation rate at approximately of 295 °C. Similar values were observed for the thermal degradation of both *P. citronellolis* mcl-PHA (Δm = 92% and T_deg_ = 295 °C) and *C. necator* P(3HB) (Δm = 97% and T_deg_ = 290 °C) (Table 2).

The P(3HB)/mcl-PHA blend produced by the co-culture presented two melting temperatures (T_m_) at 52 and 174 °C. These values are similar to the T_m_ determined for the mcl-PHA produced by *P. citronellolis* (51 °C) and to the P(3HB) produced by *C. necator* (176 °C), respectively (Table 2), which further confirms the presence of both polymers in the blend. There were two glass transition temperatures (T_g_) for the blend sample, namely at −48 and 4 °C (Table 2), which correspond to the mcl-PHA and P(3HB), respectively. The coexistence of these two thermal transitions supports the existence of a phase separation of the two polymers in the blend.

#### 3.2.5. X-Ray Diffraction 

**Figure 4:**
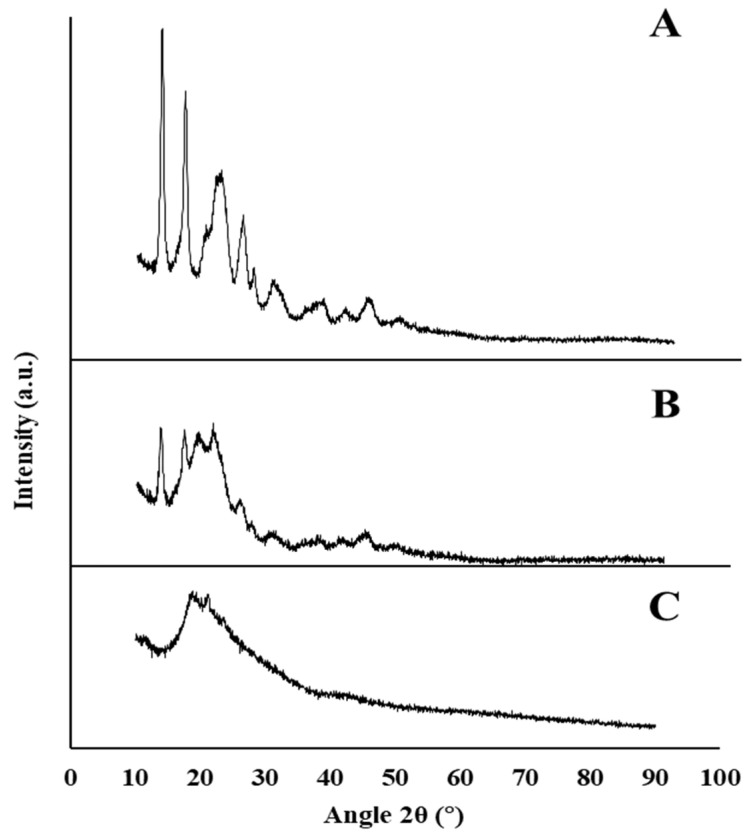
X-ray diffractogram of P(3HB) (**A**), P(3Hb)/mcl-PHA blend (**B**), and mcl-PHA (**C**) produced by *C. necator* DSM 428, the co-culture, and *P. citronellolis* NRRL B-2504, respectively, from apple pulp waste.

The XRD spectrum of the P(3HB)/mcl-PHA blend exhibited broad bands at around 2θ = 19–20°, 23° and 26°, with diffraction peaks near 14° and 17° (Figure 4B). The peaks near 14° and 17°, as well as the hump at 26°, can be ascribed to the XRD pattern of P(3HB) (Figure 4A). On the other hand, the broad hump at 19–20° can be assigned to the amorphous part of the mcl-PHA (Figure 4C). The broad hump at around 23° is probably due to the contribution of both the crystalline peak of the mcl-PHA and the amorphous phase of P(3HB).

### 3.3. Preparation and Characterization of Films Based on the Natural P(3HB)/mcl-PHA Blend 

#### 3.3.1. Morphological Characterization

**Figure 5:**
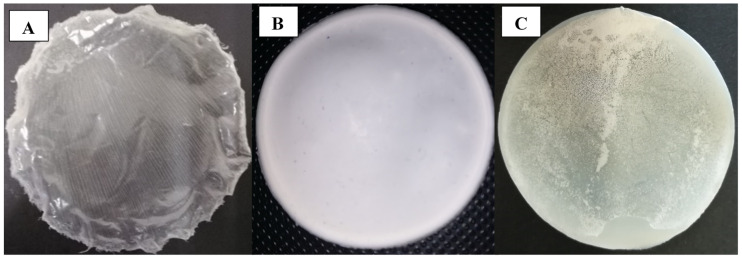
mcl-PHA (**A**), P(3HB) (**B**), and P(3HB)/mcl-PHA blend (**C**) films obtained by the solvent evaporation method (image A is reproduced with permission from Rebocho et al. [21]).

The biopolymers, namely the mcl-PHA, P(3HB) and the blend, were used to prepare films (Figure 5) by the solvent evaporation method. The mcl-PHA films thus obtained were transparent and flexible (Figure 5A), while those of P(3HB) were opaque and more rigid (Figure 5B). On the other hand, the P(3HB)/mcl-PHA blends films were heterogeneous, showing translucid and opaque areas (Figure 5C). This decrease in opacity of the polymer blend film compared to the P(3HB) film is in agreement with the thermal properties calculated from DSC. Indeed, the blend film, although not transparent, is less white than the film made only from P(3HB). Accordingly, the blend gave rise to polymer/polymer phase separation and to a partial crystallization of the two polymeric partners, but the extent of crystallization and crystal size should be higher in the case of P(3HB). The more transparent behavior of the mcl-PHA could originate from the lower size of its crystals, and then to a lower ability to scatter visible light. Nevertheless, blend films were resistant and flexible when handled, apparently combining the properties of P(3HB) and mcl-PHA.

**Figure 6:**
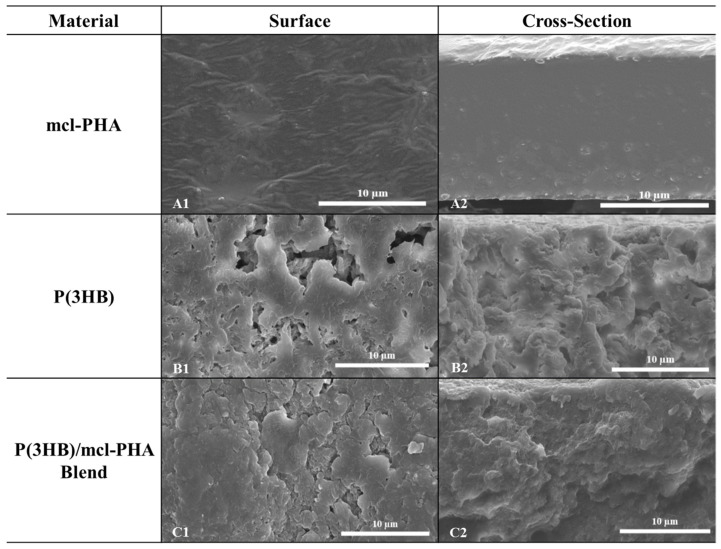
Images of the films surface layer (**left**) and cross section (**right**) assessed by scanning electron microscopy using an energy dispersive spectroscope (SEM-EDS): mcl-PHA (**A**), P(3HB) (**B**), and P(3HB)/mcl-PHA blend (**C**) films obtained by solution casting and solvent evaporation (images A1 and A2 are reproduced with permission from Rebocho et al. [21]).

SEM analysis shows the P(3HB)/mcl-PHA blend films had a rough and irregular surface (Figure 6(C1)). The films’ cross-section revealed an irregular structure with visible holes but devoid from interconnected pores (Figure 6(C2)). These observations are contrasting with those of the mcl-PHA films that were compact and had a homogeneous rough surface (Figure 6(A1)) [21]. On the other hand, the P(3HB) films disclosed a very irregular surface with visible holes, and a heterogeneous structure (Figure 6(B1,B2)), similar to the blend films. These differences in the morphology of the three films are directly in correlation with their thermal characteristics and their optical appearance discussed above.

#### 3.3.2. Swelling and Contact Angle of PHA Films 

The films showed no significant change in mass or volume during immersion in deionized water, exhibiting only negligible swelling degrees (below 5%) (Table 3), which is in agreement with their hydrophobic nature [37]. This was also confirmed by the films’ contact angles, which were above θ = 90°, characteristic of PHA hydrophobic materials [38].

The P(3HB)/mcl-PHA blend films presented a surface contact angle of 98.0 ± 0.8° (Table 3), which is close to that obtained for the mcl-PHA films (101.0 ± 0.9°) and higher than the value obtained for the P(3HB) films (79.0 ± 1.6°). The lower contact angle obtained for the P(3HB) films may be explained by their irregular rough surface as observed by the SEM analysis, which probably created a larger solid–liquid interface area, thus contributing to an increased wettability of the surface. Nevertheless, the films’ wettability needs to be improved in order to render them suitable for some specific applications, such as, for example, wound dressings that are required to maintain a moist environment. This could be achieved from an optimization of the processing parameters of film preparation.

#### 3.3.3. Gas Permeation

The permeability of the mcl-PHA/P(3HB) blend films was 2.6 and 32 Barrer for O_2_ and CO_2_, respectively (Table 3). These values are close to those obtained for *C. necator* P(3HB) film (4.2 and 26 Barrer for O_2_ and CO_2_, respectively), although the permeability to O_2_ was slightly higher for these films. On the other hand, the mcl-PHA/P(3HB) blend films were less permeable to the tested gases than the mcl-PHA films, especially regarding the permeability for O_2_ that was an order of magnitude lower (Table 3). Nevertheless, all the tested films, mcl-PHA, P(3HB), and mcl-PHA/P(3HB) blend, presented higher O_2_ permeability than the values reported for other PHAs’ films, such as, for example, P(3HB) films (0.03 Barrer) [39], P(HBHV) films (0.21 Barrer) [39], and mcl-PHA films (3.00 Barrer) [22], as well as some petroleum-based thermoplastics, such as polyethylene terephthalate (PET) (0.0004 Barrer) [40] or polystyrene (1.2 Barrer) [39,40,41]. The same can be observed for CO_2_ permeability, as the films’ permeability was higher than the values reported for P(HBHV) (2.4 Barrer) [42], PET (0.0011 Barrer) [40], Low-density polyethylene (LDPE) (0.126 Barrer) [40], and mcl-PHA (89 Barrer) [22].

#### 3.3.4. Mechanical Properties 

The P(3HB)/mcl-PHA blend films (Table 3) presented a tensile strength at break of 1.47 ± 0.70 MPa, with a deformation of 338 ± 19 % upon breaking, associated with a Young modulus of 5.42 ± 1.02 MPa, demonstrating the films were susceptible to elastic and plastic deformations. Taking these values into account, the P(3HB)/mcl-PHA films revealed more similarity to mcl-PHA films from *P. citronellolis* than the P(3HB) films from *C. necator* (Table 3). The decrease in the tensile strength and the Young Mmodulus have been suggested in blends of poly(hydroxybutyrate-*co*-hydroxyvalerate (PHBHV) with mcl-PHA by solution blending [13]. However, in the results reported, the content of mcl-PHA above 5 wt% led to a decrease in the strain at break, which did not happen for the P(3HB)/mcl-PHA blend films tested in this study.

The P(3HB)/mcl-PHA blend films were found to be more prone to deformation and to be much less rigid than P(3HB) films, which demonstrated a strain at break of 10 ± 2%, Young modulus of 583.78 ± 32.05, and tensile strength at break of 19.28 ± 1.13 (Table 3). Those mechanical properties are directly in line with the thermal features of these materials. In particular, the decrease in crystallinity of the two polymers arising from their blending can easily explain the deformation ability evidenced by P(3HB)/mcl-PHA films. In the literature, similar results are described for blends of PHBHV and natural rubber when compared to PHBHV films alone. When natural rubber was blended with PHVHB, their films become more prone to deformation, with a corresponding reduction in the maximal tensile strength [9].

## 4. Conclusions

Apple pulp waste from the fruit processing industry was successfully used as the sole carbon source for the co-culture of *C. necator* DSM 428 and *P. citronellolis* NRRL B-2504 to produce a natural P(3HB)/mcl-PHA blend. The co-culture approach was effective in improving the substrate consumption efficiency compared to the individual strains’ monocultures. The obtained P(3HB)/mcl-PHA blend, composed of nearly equal contents of each biopolymer, was processed into flexible and elastic films that combined the properties of both P(3HB) and mcl-PHA. The films’ mechanical properties were similar to mcl-PHA films, while their permeabilities to O_2_ and CO_2_ were comparable to those of P(3HB). Although the P(3HB)/mcl-PHA blend films’ properties still require tuning to make them suitable for specific uses, such as, for example, biomedical applications, their potential was demonstrated and supports further works on their development.

## Figures and Tables

**Table 1:** Kinetic and stoichiometric parameters of the three batches produced by *P. citronellolis* NRRL B-2504, *C. necator* DSM 428, and the co-culture using apple pulp waste (CDM, cell dry mass; X, rest biomass; *r_p_*, volumetric productivity).

Parameter	Monoculture		Co-Culture
	*P. citronellolis* NRRL B-2504	*C. necator* DSM 428	*P. citronellolis* NRRL B-2504 and *C. necator* DSM 428
µ_max_ (h^−1^)	0.24 ± 0.01	0.14 ± 0.05	0.23 ± 0.02
CDM (g/L)	4.00 ± 0.08	6.93 ± 0.09	5.51 ± 0.09
X (g/L)	2.80 ± 0.06	3.90 ± 0.05	3.66 ± 0.06
PHA (wt%)	30.0 ± 1.7	43.7 ± 2.5	33.6 ± 1.9
PHA (g/L)	1.20 ± 0.05	3.03 ± 0.04	1.85 ± 0.03
*r*_p_ (g/(L·h))	0.025 ± 0.001	0.066 ± 0.003	0.040 ± 0.002
Sugars consumption (g/L)	10.03 ± 1.43	6.67 ± 0.25	17.02 ± 0.76
Fructose	2.50 ± 0.92	4.07 ± 0.17	6.59 ± 0.76
Glucose	7.53 ± 0.51	3.06 ± 0.08	9.10 ± 1.08
Sucrose	0.00	0.00	1.34 ± 0.32
References	[21]	This study	This study

**Table 2:** Monomeric composition of the polymers’ samples extracted from the biomass of *P. citronellolis* and/or *C. necator* (3HB, 3-hydroxybutyrate; 3HHx, 3-hydroxyhexanoate; 3HO, 3-hydroxyoctanoate; 3HD, 3-hydroxydecanoate; 3HDd, 3-hydroxydodecanoate; 3HTd, 3-hydrotetradecanoate; M_w_, molecular weight; PDI, polydispersity index; T_g_, glass transition temperature; T_m_, melting temperature; T_deg_, degradation temperature; ΔHm, melting enthalpy).

Parameter	Monocultures		Co-Culture
	*P. citronellolis* NRRL B-2504	*C. necator* DSM 428	*P. citronellolis* NRRL B-2504 and *C. necator* DSM 428
Composition (wt%)			
3HB	0	100	48
3HHx	1	0	0.5
3HO	22	0	10
3HD	68	0	35
3HDd	5	0	3
3HTd	4	0	3
Mw (×10^5^ Da)	3.7	5.0	4.3
PDI	2.1	2.0	2.2
T_g_ (°C)	n.d.	n.d.	−48/4
T_m_ (°C)	51	176	52/174
T_deg_ (°C)	296	293	297
Reference	[21]	This study	This study

n.d.: not detected.

**Table 3:** Characteristics of the films prepared from mcl-PHA, P(3HB), and P(3HB)/mcl-PHA blend: water contact angles, permeability to gases, and mechanical properties.

Parameter	mcl-PHA	P(3HB)	P(3HB)/mcl-PHA Blend
Swelling in water (%)	2	4	5
Water Contact Angle (θ)	101.0 ± 0.9	79.0 ± 1.6	98.0 ± 0.8
Permeability (Barrer)			
O_2_	11 ± 0.05	4.2 ± 0.05	2.6 ± 0.05
CO_2_	53 ± 0.05	26 ± 0.05	32 ± 0.05
Tensile strength at break (MPa)	4.86 ± 0.68	19.28 ± 1.13	1.47 ± 0.70
Deformation at break (%)	279 ± 12	10 ± 2	338 ± 19
Young Modulus (MPa)	7.80 ± 1.58	583.78 ± 32.05	5.42 ± 1.02
References	[21]	This study	This study
